# Oral Health and nutritional status in nursing home residents—results of an explorative cross-sectional pilot study

**DOI:** 10.1186/s12877-017-0429-0

**Published:** 2017-01-31

**Authors:** Dirk Ziebolz, Christine Werner, Gerhard Schmalz, Ina Nitschke, Rainer Haak, Rainer F. Mausberg, Jean-François Chenot

**Affiliations:** 1 Department of Cariology, Endodontology and Periodontology, University of Leipzig, Liebigstr. 12, D 04103 Leipzig, Germany; 2 Dental practice, Goettingen, Germany; 3 Department of Prosthodontics and Materials Science, University of Leipzig, Leipzig, Germany; 4 Deptartment of Preventive Dentistry, Periodontology and Cariology, University University Medical Center, Goettingen, Germany; 5 Department of General Practice, Institute for Community Medicine, University Medicine Greifswald, Greifswald, Germany; 6 Clinic of Geriatric and Special Care Dentistry, Center of Dentistry, University of Zurich, Zurich, Switzerland

**Keywords:** Oral health, Nutritional status, Nursing home, Dementia

## Abstract

**Background:**

This study was performed to assess oral and nutritional status of nursing home residents in a region of Lower Saxony, Germany. The aim was to show potential associations between oral status (dentate or edentulous), further anamnestic factors (dementia, age, smoking) and the risk for malnutrition in this population.

**Methods:**

In this observational cross-sectional pilot study of residents from four nursing homes Mini Nutritional Assessment (MNA), Body-Mass-Index (BMI), dental status (DMF-T) and periodontal situation (PSR^®^/PSI) were recorded. Associations of recorded factors with oral health and nutritional status were examined in univariate and multivariate analysis.

**Results:**

Eighty-seven residents participated in the study (mean age: 84.1 years; female: 72%, demented: 47%). Average BMI was 26.2 kg/m^2^; according MNA 52% were at risk for malnutrition. 48% of the residents were edentulous, and the average DMF-T of dentulous was 25.0 (3.7) (D-T: 2.0 [3.1], M-T: 15.0 [8.3], F-T: 8.0 [7.4]); PSR^®^/PSI 3 and 4 (need for periodontal treatment) showed 79% of residents. In univariate analysis dementia (OR 2.5 CI_95_ 1.1–5.6) but not being edentulous (OR 2.0 CI_95_ 0.8–5.8) were associated with being at risk for malnutrition. Dementia remained associated in multivariate analysis adjusting for age and sex, (OR 3.1 CI_95_ 1.2–8.2) and additionally being edentulous (OR 2.8 CI_95_ 1.1–7.3) became associated significantly. Furthermore, nursing home residents with dementia had more remaining teeth (OR 2.5 CI_95_ 1.1–5.9).

**Conclusion:**

Dementia was a stronger predictor for risk of malnutrition in nursing home residents than being edentulous. Further studies to elucidate the possible role of oral health as cofactor for malnutrition in dementia are needed.

## Background

Oral health situation of nursing home residents is often poor due to age and limited access to dental care. This might be a symptom as well as a cause of poor general health and nutritional status [1, 2]. Due to demographic and societal changes it is expected that the number of elderly people in nursing homes will increase [3]. Although the number of edentulous people is increasing with age, many of these nursing home residents have remaining teeth [4]. This might be due to advances in prevention and improved oral hygiene [5]. Corresponding developments have already been shown for elderly German individuals in the fourth German oral health study [6]. Many nursing home residents are unable to practice oral hygiene because of physical or cognitive disabilities. However, the oral health care support in nursing homes is often limited resulting in poor oral hygiene and oral health [7, 8]. These hygienic deficits affect not only teeth but also prostheses [9]. Furthermore, elderly nursing home residents are partly unable to communicate their oral health problems to nursing staff or show defensiveness [10]; so most interventions are only carried out if there is pain [10].

Moreover, nutrition has an important role in quality of life, especially among the elderly, and food intake has a tremendous influence on morbidity and mortality [11]. There is evidence that poor oral health, xerostomia and reduced chewing ability cause involuntary weight loss in the elderly population [12]. In this context, oral health and dental status have an influence on the nutritional status of residents [13]. Tooth loss, in particular, leads to loss of chewing function and thus, causes residents’ avoidance of food, as it is difficult to chew foods like fruits and vegetables [14]. In this manner, poor oral status and insufficient prosthetic restorations might be one of the important risk factors for malnutrition in nursing homes.

However other risk factors for malnutrition in older adults are depression, cognitive impairment, dementia, functional impairment, and swallowing difficulty [15, 16]. Dementia and also cognitive impairment might also have an influence on oral conditions. Accordingly, demented nursing home residents show higher periodontal treatment need [17, 18]. Especially the association between cognitive impairment and tooth loss has been discussed, showing heterogeneous results [19–22]. Although the fifth German oral health study (DMS V) has investigated nursing home residents for the first time in a representative population study in Germany [23], only few studies on oral health in this patient group in Germany are available [1, 17]. It was hypothesized that both, dementia and being edentulous are predictors for the risk for malnutrition. Furthermore, the relationship of oral health and other factors like dementia in nursing home residents should be explored.

Therefore, this study was performed to assess oral health (dental and periodontal condition) and nutritional status of nursing home residents in a region of Lower Saxony, Germany. The aim was to investigate potential associations between oral status (dentate or edentulous), further anamnestic factors (dementia, age, smoking) and the risk for malnutrition in this population.

## Methods

This was a observational cross sectional pilot study with elderly individuals dwelling in nursing homes in Northern Germany conducted between March and August 2011. This study was reviewed and approved by the ethics committee of the University Medical Center Goettingen, Germany (No. 21/3/10). Nursing home residents or their legal guardian were informed verbally and in writing about the study and gave written informed consent.

### Participants

A total of 370 elderly individuals living in four different nursing homes in south lower Saxony (area of Goettingen and Northeim, Germany) were invited to participate in the study. All residents of the nursing homes respectively their legal guardians, mostly relatives, were asked to participate. The inclusion criteria were: age; older than 55 years and living in nursing homes in the area of Goettingen and Northeim and written informed consent by nursing home residents themselves of their legal guardian. Exclusion criteria were: feeding through percutaneous endoscopic gastrostomy tube (PEG tube) and the inability to cooperate for the oral examination.

#### Recording of subject data

Participants’ health data were obtained from resident’s health record of the nursing home. The following aspects were recorded: age, gender, bedridden (yes or no), diagnosed dementia (yes or no), weight and height and active smoking (yes or no). No additional tests, e.g. to grade the severity of dementia, were performed.

### Assessment of nutritional condition

The nutritional condition was assessed with the “Mini Nutritional Assessment “(MNA^®^) by the same examiner for all patients (CW) [24, 25]. The MNA consist of simple measurements (weight, height, and weight loss) and brief questions; related to lifestyle, medication, and mobility and diet) and subjective assessment. The maximum score is 30, a score ≥ 24 indicates adequate nutritional status, a score between 17 and 23.5 risk of malnutrition and score <17 malnourishment.

### Dental examination

All subjects were examined once by an experienced dentist (CW) with a headlight in the room of the participants. The dental examination included dental status (DMF-T) and assessment of periodontal treatment need (PSR^®^/PSI).

- *Dental findings/status (DMF-T)* [26]: The DMF-T is a caries index to quantify the number of decayed teeth, missing teeth and filled teeth with values ranging from 0 to 28 without wisdom-teeth. The DMF-T was assessed visually with mirror and probe. Based on the number of decayed, missing, and filled teeth, the DMF-T was determined as follows: All teeth with a reasonable suspicion of/or definitely showing a cavity in the dentine layer, were assigned to the D (=decayed) component; filled and crowned teeth were evaluated and assigned to component F (=filled); missing teeth were assigned to the M component (=missing).
- *Periodontal screening (PSR* ^*®*^ */PSI)* [27]: Periodontal treatment need was determined by probing depth of periodontal pocket and by extend of the resulting bleeding. The periodontal situation (periodontal treatment need) was evaluated with the periodontal screening index (PSR^®^/PSI). The examination was performed with the WHO probe (Morita, Kyoto, Japan) at 6 points per tooth and the PSR^®^/PSI score was recorded using the following criteria:
  - ➢PSR^®^/PSI 0: if pocket depth < 3.5 mm, no bleeding and no calculus.
  - ➢PSR^®^/PSI 1: if pocket depth < 3.5 mm, bleeding on probing and no calculus.
  - ➢PSR^®^/PSI 2: if pocket depth < 3.5 mm, bleeding on probing and calculus is present.
  - ➢PSR^®^/PSI 3: if pocket depth is 3.5–5.5 mm.
  - ➢PSR^®^/PSI 4: if pocket depth is > 5.5 mm.

Maxilla and mandible were divided in 3 sextants: 1 of the anterior and 2 of the posterior teeth. The highest score was determined for each sextant of the dentition. PSR^®^/PSI scores between 3 and 4 stated for need for periodontal treatment.

### Statistical analysis

Descriptive statistics of data are reported as the means ± standard deviation (SD) or median and range. For univariate inferential statistical analyses Chi^2^-test or Fisher’s exact test for categorical data and *t*-test or *U*-Test for continuous data were used. For multivariate analysis logistic regression covariates were chosen based on a reasonable frequency (at least 20% in each group), including age and gender based on a recommendation from Allison [28]. We did not include data from the MNA since the predicted outcome, being at risk for malnutrition, was based on the items form the MNA. The goodness of fit was assessed with the Hosmer Lemeshow test and for the presence of multicollinearity the Bravais-Pearson correlation coefficient was used. The software package SAS 9.4 was used for analysis.

## Results

### Subjects

A total of 100 nursing home residents out of 370 contacted residents in four nursing homes agreed to participate in the study. Only 90 could be examined and nutritional assessment could only be performed in 87 participants with a mean age of 84 years (8.6) [median: 85.9]. Most common reason for non-participation was the inability to obtain informed consent from patients with legal guardians. Most subjects were preadipose according to WHO [29], respectively normal findings in accordance to adaptation for geriatric patients [30] with a mean BMI of 26.2 (5.0) kg/m^2^ [median: 25.1]. 55% (*n* = 48) of the investigated residents had a diagnosed dementia according to medical records. Five subjects were totally bedridden (Table 1).

**Table 1:** Subject characteristics [*n* = 87] based on chart review and interview with care giving nurse

Age in years mean (SD) [median; range]		84.1 (8.6) [85.9; 56–102]
Gender	Female	68 (78%)
	Male	19 (22%)
Current smoker	Non-smoker	75 (86%)
	Smoker	12 (14%)
Body-mass-index in kg/m^2^ mean (SD) [median; range]		26.2 (5.0) [25.2; 21.8–29.0]
Dementia (yes/no)	No	39 (45%)
	Yes	48 (55%)
Totally bedridden	No	82 (94%)
	Yes	5 (6%)

*Abbreviations*: *SD* standard deviation

### Nutritional condition (MNA)

The median value of the MNA screening was 11. About half (52%) were at risk for malnutrition according to MNA, nobody was judged as being malnourished (Table 2). Of the 45 subjects at risk of malnutrition 80% took more than three medications and 22% needed help with meals.

**Table 2:** Results of the MNA (Mini Nutritional Assessment) screening of the participants [*n* = 87]

Declined food intake over the past 3 months ^a^	Severe decrease	1 (1%)
	Moderate decrease	19 (21%)
	No decrease	67 (78%)
Weight loss during the past 3 months ^a^	>3 kg	5 (6%)
	1–3 kg	14 (17%)
	no	65 (74%)
	Unknown	3 (3%)
Mobility ^a^	Bed of chair bound	21 (24%)
	Able to get out of bed / chair but does not go out	17 (20%)
	Goes out	49 (56%)
Psychological stress or acute disease ^a^	No	72 (83%)
	Yes	15 (17%)
Neuropsychological problems ^a^	No problems	39 (45%)
	Mild dementia	26 (30%)
	Severe dementia or depression	22 (25%)
Body-mass-index	<19	2 (2%)
	19–21	11 (13%)
	21–23	19 (22%)
	>23	55 (63%)
MNA screening points (median and IQR)		11 (9–13)
Risk for malnutrition	Normal nutritional status (12–14 MNA screening points)	42 (48%)
	At risk of malnutrition (8–11 MNA screening points)	45 (52%)

Only the first part of MNA was performed for all patients and is therefore shown in Table 2. The second part of MNA was only executed for residents with (risk for) malnutrition

*Abbreviations*: *IQR* interquartile range

^a^ as judged by the care giving nurse

### Dental (DMF-T) and periodontal findings (PSR®/PSI)

A total of 43 (48%) of the participants, which allowed oral examination were toothless, while 47 (52%) had remaining teeth.

The mean DMF-T of all participants was 26.4 (3.1). On average, 1.1 (2.4) teeth were carious (D-T) and 4.1 (6.5) filled (F-T); the average M-T value was 21.2 (8.8). The mean DMF-T of residents (*n* = 47) with teeth was 25.0 (3.7), showing M-T-values of 15.0 (8.3) (mean D-T: 2.0 [3.1], mean F-T: 8.0 [7.4]); 45% participants out of these subjects have 14 or more teeth (Table 3).

**Table 3:** Oral health of the participants [*n* = 87]

Parameter			Mean (SD) [median]
All participants (*n* = 87)	DMF-T index		26.4 (3.1) [28]
	Decayed teeth		1.1 (2.4) [0]
	Missing teeth		21.2 (8.8) [26,5]
	Filled teeth		4.1 (6.5) [0]
	Edentulous residents		*n* = 41 (47%)
Residents with teeth (*n* = 46)	DMF-T index		25.0 (3.7) [27]
	Decayed teeth		2.0 (3.1) [1]
	Missing teeth		15.0 (8.3) [15]
	Filled teeth		8.0 (7.4) [6]
	Remaining teeth (≥14 teeth)		*n* = 21 (45%)
	PSI / PSR [*n* = 38]	Score 1	1 (3%)
		Score 2	7 (18%)
		Score 3	16 (42%)
		Score 4	14 (37%)

*Abbreviations*: *SD* standard deviation

The periodontal situation could be assessed of 38 nursing home residents. No participant with teeth was periodontal healthy (PSR®/PSI score 0). Eight subjects (21%) had a highest PSR®/PSI score of 1 or 2, 16 subjects (42%) had a PSR®/PSI score of 3, while 14 subjects (37%) had a PSR®/PSI score of 4. According to the definition, 79% of the periodontal investigated dentate patients have a periodontal treatment need (Table 3).

### Association of dentition and nutritional status

In univariate analysis beside diagnosed dementia (OR 2.5 CI_95_ 1.1–5.6) the items from MNA (mobility, neuropsychological problems and BMI) were significantly associated with being at risk for malnutrition, in contrast to edentulous (OR 2.0 CI 0.8–5.8) or other factors (Table 4).

**Table 4:** Comparison of patients not at risk and at risk of malnutrition regarding general and oral parameters

Risk factors	Nutritional status		Univariate analysis			Multivariate analysis ^a^	
	Not at risk of malnutrition (12–14 MNA screening points) *n* = 42 (48%)	At risk of malnutrition (8–11 MNA screening points) *n* = 45 (52%)	OR	*p*-value		OR	*p*-value
General parameters							
Age in years [mean (SD)]	83.1 (9.3)	84.9 (7.3)	n.a.	0.33		1.0 (CI_95_ 0.9–1.1)	n.s.
Gender (female)	31 (46%)	37 (54%)	1.1 (CI_95_ 0.4–2.7)	0.81		1.2 (CI_95_ 0.4–3.8)	n.s.
Smoking status (yes)		6 (50%)	6 (50%)	0.9 (CI_95_ 0.3–3.1)	0.86	0.8 (CI_95_ 0.2–3.1)	n.s
Dementia according to medical records (yes)		17 (40%)	31 (60%)	2.5 (CI_95_ 1.1–5.6)	0.03	3.1 (CI_95_ 1.2–8.2)	0.02
Permanently bedridden according to medical records (yes)		1 (2%)	4 (9%)	4.0 (CI_95_ 0.4–37)	0.19	n.a.	n.a.
Neuropsychological problems according to MNA	No problems	26 (62%)	13 (29%)	n.a.	<0.0001	n.a.	n.a.
	Mild dementia	14 (17%)	12 (27%)				
	Severe dementia or depression	2 (2%)	24 (44%)				
Mobility according to the MNA	Bed of chair bound	1 (2%)	20 (44%)	n.a.	<0.0001	n.a.	n.a.
	Able to get out of bed / chair but does not go out	8 (19%)	9 (20%)				
	Goes out	33 (79%)	16 (36%)				
Body-mass-index kg/m^2^ [mean (SD)]		27.7 (4.5)	24.7 (5.1)	n.a.	0.005	n.a.	n.a.
Oral parameters							
Edentulous		16 (38%)	25 (56%)	2.0 (CI_95_ 0.8–5.8)	0.1	2.8 (CI_95_ 1.1–7.3)	0.03
Remaining teeth		26 (62%)	20 (44%)				
DMF-T (only those with teeth *n* = 46)		25.6 (2.9)	24.5 (4.3)	n.a.	0.34	n.a.	n.a.
PSI / PSR score (*n* = 37)	max. Score 1 or 2	5 (24%)	3 (19%)	1.3 (CI_95_ 0.4–6.7)	0.71	n.a.	n.a.
	max. Score 3 or 4	16 (76%)	13 (81%)				

*Abbreviations*: *OR* odds ratio, *CI*_*95*_ confidence interval, *SD* standard deviation, *MNA* (mini nutritional assessment), *n.a.* not applicable, *n.s.* not significant

^a^ Hosmer Lemeshow-test indicated goodness of fit (chi^2^ 0.37) and multicollinearity was excluded

Dementia remained associated in multivariate analysis adjusting for age and sex, (OR 3.1 CI_95_ 1.2–8.2) and additionally being edentulous (OR 2.8 CI_95_ 1.1–7.3) became associated significantly (Table 4). Among those with teeth no relationship between oral health situation (DMFT or PSR®/PSI) and risk of malnutrition was observed (Table 4). Factors associated with toothlesness are shown in Table 5. Nursing home residents with dementia were more likely to have remaining teeth (OR 2.5 CI_95_ 1.1–5.9; Table 5).

**Table 5:** Univariate analysis of association of dental status (edentulous vs. dentulous) on different factors including age, body-mass index (BMI), risk for malnutrition, gender, smoking and dementia

Parameter		Edentulous [*n* = 41]	Dentulous [*n* = 46]	OR (CI_95_)	*p*-value
Age in years [mean (SD)]		84.8 (8.3)	83.6 (8.4)	n.a.	0.51
Body-mass-index kg/m^2^ [mean (SD)]		26.0 (4.8)	26.3 (5.2)	n.a.	0.97
Risk for malnutrition	No [*n* = 42]	16 (38%)	26 (62%)	2.0 (0.8–5.8)	0.1
	Yes [*n* = 45]	25 (56%)	20 (44%)		
Gender	Female [*n* = 68]	33 (49%)	35 (51%)	1.9 (0.8–5.0)	0.16
	Male [*n* = 19]	8 (42%)	11 (58%)		
Smoking status	No [*n* = 75]	35 (47%)	40 (53%)	0.9 (0.2–3.0)	0.86
	Yes [*n* = 12]	6 (50%)	6 (50%)		
Dementia	No [*n* = 39]	24 (62%)	15 (38%)	2.5 (1.1–5.9)	0.03
	Yes [*n* = 48]	17 (36%)	31 (64%)		

*Abbreviations*: *CI*_*95*_ 95% confidence interval, *n.a.* not applicable, *OR* odds ratio, *SD* standard deviation

## Discussion

### Summary of the main results

A weak relationship between toothlessness and being at risk for malnutrition in nursing home residents was observed, but only became apparent after adjusting for other factors. Among those with remaining teeth the dental status (decayed and missing teeth) and periodontal health was not associated with an increased risk for malnutrition. The strongest observed risk factor was a record of diagnosed dementia. Patients with dementia had more often remaining teeth compared to those without.

### Strengths and limitations

This is one of few studies on oral health of elderly nursing home residents in Germany including demented and bedridden residents. It was difficult to obtain informed consent from legal guardians and perform the oral examination. Therefore we have low participation rate (27%) of 100 from 370 potentially eligible participants of whom only from 87 all data could be obtained. This might have introduced selection bias and threaten the generalizability of the current study’s findings. Although a multivariate analysis for the main outcome nutritional status was conducted, the number of subjects precludes more extensive analysis. It must be also kept in mind that this is a cross sectional study, especially relating to temporality pertaining to the presence of teeth and dementia. The amounts of collected data pertinent for malnutrition were limited by lack of resources. For the diagnosis of dementia we relied on medical records and we did not attempt to assess the severity of dementia. It is known, that oral health deficiencies are more present in severe cognitive impairment [31]. Other possible risk factors for malnutrition like depression, functional impairment, difficulties swallowing, co-morbidities and medication were not collected [15].

### Comparison with existing literature

In comparing the results of the current study with the fourth and fifth German oral health study (DMS IV and V), a representative study for German general population, similar trends in DMF-T and need for periodontal treatment are apparent [6, 23]. About half (47%) of the individuals in the study (mean age: 84.1 years) were edentulous. This trend is consistent with the international literature with a prevalence range between 22 and 64% [8, 32–36]. Furthermore, in the current study, participants with remaining teeth had a high need for periodontal treatment what also corresponds to literature; prevalence range is between 26 and 84% [23, 37–39].

Furthermore, 52% of participants in the current study were at risk for malnutrition according to the MNA. Recent studies in nursing homes, which also performed the MNA, showed a higher amount of elderly with risk for malnutrition. Thereby, Amorim Sena Pereira et al. (2014) registered 66% of the evaluated elderly as malnourished or respectively at risk of malnutrition in Brazil [40]. Nazemi et al. (2014) showed that 69% were at risk of malnutrition and 10% were categorized as malnourished in Iran [41], while Gordon et al. (2014) registered that 30% of participants were malnourished. A further 56% were at risk for malnutrition in UK [42]. An earlier review article reported a prevalence range for undernutrition in institutionalized geriatric residents of between 1 and 83% [43], suggesting high variability between different nursing homes.

In the current study, being edentulous was only in multivariate analysis significantly associated to malnutrition. Other studies showed edentulous residents, especially without dentures, to be at higher risk for malnutrition [44, 45]. In this respect, however, it should be acknowledged that in the current study, the prosthetic situation of edentulous residents was not covered, which might explain why edentulous residents were not at a significantly higher risk for malnutrition. It was already discussed that an improvement in nutritional status by prosthetic treatment is achievable, showing heterogeneous results [46–48]. It seems thereby clear that tooth replacement alone is not able to prevent malnutrition [48]. In contrast, dementia was associated to malnutrition in both, multivariate and univariate analysis. This is in accordance to recent literature, where an association between dementia and malnutrition is apparent [15, 49]. It is also discussed weather malnutrition might influence the cognitive status, what might result in a higher prevalence of dementia in malnourished residents [50].

In the current study, participants with dementia were more likely to have remaining teeth. With regard to this issue there are mixed results. Several studies showed dementia to be unrelated to tooth loss [19, 20], while other studies showed cognitively impaired residents to have a higher prevalence of toothlessness [21, 22]. Consequently, the results of the current study are not in line with the recent literature. Only one study already found lower prevalence of toothlessness in demented patients [51]. This study also detected poor oral hygiene and health in patients with dementia [51]. Possible explanations for these findings might be an inadequate dental care after developing dementia; leading to a higher number of retained teeth, which are not worth preserving. Decayed or periodontal damaged teeth are not extracted due to decreased ability to complain about oral health problems. Additionally, frequently prescribed anticholinergic drugs, decreasing salivation, to control disturbing behaviors might add to the decay of teeth.

Further studies showed also a high need for periodontal treatment in elderly individuals with dementia [17, 18]. In this context, a potential correlation between dementia and periodontitis is recently discussed [52]. It might therefore be possible, that the higher number of remaining teeth in demented participants lead to a higher prevalence of dental and periodontal diseases. This could cause pain and problems, leading to difficulties during food intake, resulting in a higher risk of malnutrition. Therefore, improving oral hygiene in nursing home residents with remaining teeth and regular oral examination particularly for residents with dementia is desirable.

## Conclusion

Beside of dementia status, being edentulous was the only dental parameter, which was weakly associated with risk for malnutrition. Overall the oral health was poor, which affected particularly nursing home residents with dementia which had more remaining teeth. Further studies to elucidate the possible role of oral health as cofactor for malnutrition in dementia are needed.

## Abbreviations

BMI: Body mass index
MNA: Mini nutritional assessment
DMF-T: Decayed (D), missing (M), and filled (F) teeth (T) index
PSR®/PSI: Periodontal screening index
DMS IV: Fourth German oral health study
DMS V: Fifth German oral health study
